# Supplementary material for: Cell painting in activated cells illuminates phenotypic dark space and uncovers novel drug mechanisms of action
Source: Res Sq. 2025 Jun 23:rs.3.rs-6734784. Preprint. [Version 1] doi: 10.21203/rs.3.rs-6734784/v1 (PMC12270227; doi:10.21203/rs.3.rs-6734784/v1)
Supplement: Supplement 1 [file NIHPPrs6734784v1-supplement-1.pdf]

Supp.Figure 1

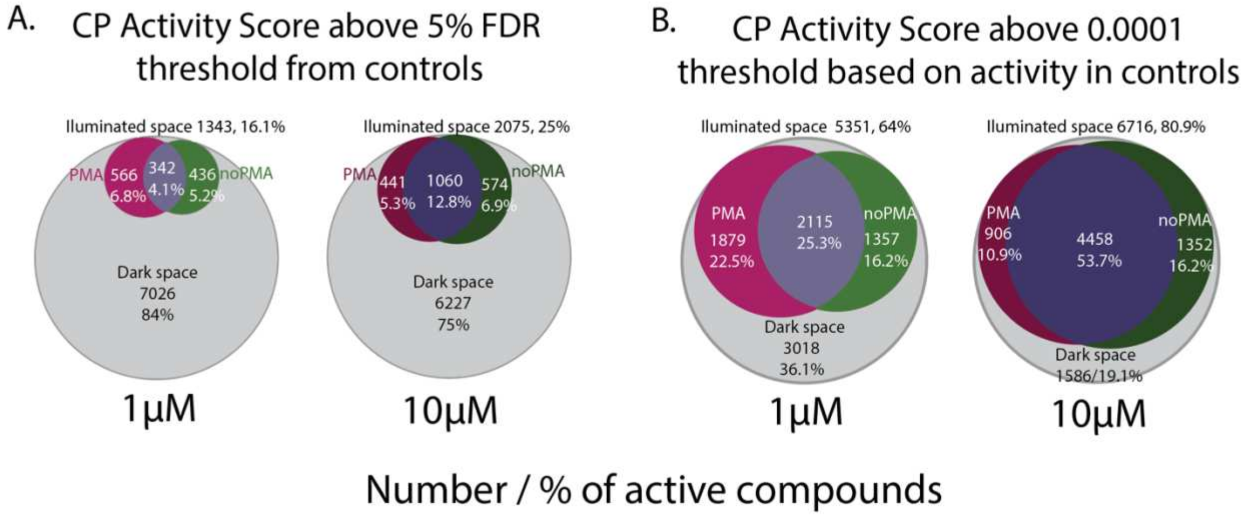

Supp.Figure 1. Drawing the cutoff for compound activity is a balancing act between tolerating false positives and accepting false negatives; here, we present a couple of methods in addition to IRQ used in a main text (Fig.4C,E)

A. The illuminated phenotypic space ranges from 16 to 25%, depending on compound concentration if 5% FDR (False Discovery Rate) hit calling method is used (see Methods and Fig.4E) **B.** If the activity threshold is established just above the inflection point of an elbow plot (0.0001) (see Fig.4E), then the percentage of compounds with significant effect ranges from 64 to 80%

Supp. Method

Pathway Enrichment Analysis Methodology

Data Preprocessing and Filtering

The analysis began with preprocessing of UMAP (Uniform Manifold Approximation and Projection) data from high-content screening of compounds under different treatment conditions (PMA and noPMA) at two concentrations (1 µM and 10 µM). The analysis pipeline consisted of the following steps:

1. Initial Data Selection: The umap_filtering.py script filtered UMAP data from original high-dimensional phenotypic profiles. Compounds were matched with their target annotations from key files containing threshold CP scores above 1e-4, ensuring only compounds with significant activity were included. The data was organized into four primary datasets: PMA 1 µM, PMA 10 µM, noPMA 1 µM, and noPMA 10 µM.

2. Target Filtering: The filter_targets.py script further refined the datasets by keeping only targets represented by at least 3 compounds, excluding the generic "Others" category. This ensured statistical robustness in subsequent analyses by focusing on well-represented targets. The filtering resulted in distinct sets of compounds for each condition, with detailed target distribution statistics saved for reference.

Clustering and Entropy Calculation

The entropy analysis was performed using the target_entropy_analysis.py script through the following steps:

1. UMAP-Based Clustering: The script loaded filtered UMAP data for each condition and concentration. For each dataset, two clustering methods were applied:
    - K-Means clustering with 50 clusters
    - HDBSCAN (Hierarchical Density-Based Spatial Clustering of Applications with Noise) with a minimum cluster size of 10

2. Target Entropy Calculation: For each target across the datasets, Shannon entropy was calculated to quantify the spread of compounds belonging to that target across different clusters. Lower entropy values indicate higher consolidation (compounds are concentrated in fewer clusters), while higher entropy values indicate dispersal (compounds are spread across many clusters). Only targets with at least 5 compounds were included in this analysis to ensure statistical reliability.

3. Entropy Comparison: The script generated entropy comparison plots and data files (entropy_comparison_1uM.csv and entropy_comparison_10uM.csv), which captured the differences in entropy values between PMA and noPMA conditions for each target at each concentration. A negative entropy difference (PMA - noPMA) indicated greater consolidation of

a target in the PMA condition, while a positive difference indicated greater consolidation in the noPMA condition.

Pathway Enrichment Analysis

The pathway enrichment analysis was conducted using the analyze_pathway_enrichment.py script:

1. Target Categorization: Targets were categorized into specific pathway groups based on biological function using a comprehensive classification system. The classification covered major categories including:
    - Neurotransmitter systems (Serotonergic, Dopaminergic, etc.)
    - Ion channels and transporters
    - Kinase subfamilies
    - Nuclear receptor subfamilies
    - GPCRs (G-protein coupled receptors)
    - Various enzyme types
    - Metabolic pathways
    - Cytoskeleton and cellular processes
    - Cell death and survival mechanisms
    - Immune and inflammatory systems
    - Epigenetic regulation
    - Redox systems
2. Pathway Distribution Analysis: The script analyzed the distribution of pathway categories among targets showing significant entropy differences between conditions. It identified pathways that were preferentially consolidated in either PMA or noPMA conditions.
3. Enrichment Calculation: Enrichment scores were calculated as the ratio of the percentage representation of each pathway category in PMA-consolidated targets versus noPMA-consolidated targets. This quantified which biological systems showed the strongest condition-dependent consolidation effects.

Network Visualization

The visualize_cluster_networks.py script was used to create network visualizations that further illustrated the relationships between clusters and targets:

1. Network Construction: Clusters were represented as nodes in a network, with edges connecting clusters based on their proximity in UMAP space. Each cluster was colored based on its most enriched targets, with size proportional to target enrichment.

2. Target Distribution Analysis: For each condition and concentration, target distribution across clusters was analyzed and visualized as heatmaps, providing another perspective on target consolidation patterns.

3. Integrated Visualization: Comprehensive visualizations were created combining UMAP scatter plots, zoomed views of specific clusters, and network representations to highlight the most interesting patterns of target consolidation.

The analysis revealed significant differences in target consolidation patterns between PMA and noPMA conditions, with specific biological pathways showing preferential consolidation in each condition. These findings provided insights into how pathway activation patterns differ under PMA treatment, which activates protein kinase C signaling pathways, and contributes to our understanding of biological system organization in response to different cellular states.

Shannon Entropy Calculation

The Shannon entropy for each target's distribution across clusters was calculated using the following equation:

$$H(T) = -\sum_{i=1}^{N} \quad p_i(p_i)$$

Where:

- *H(T)* is the entropy value for target T
- p_i is the probability of finding a compound with target T in cluster i
- *N* is the total number of clusters where the target appears
- log_2 indicates the base-2 logarithm, so entropy is measured in bits

In the implementation, the probabilities were calculated by:

$$p_i = \frac{n_{i,T}}{\sum_{j=1}^{N} \quad n_{j,T}}$$

Where:

- $n_{i,T}$ is the number of compounds with target T in cluster *i*
- The denominator is the total number of compounds with target *T* across all clusters

Entropy Difference Calculation

The comparison between PMA and noPMA conditions was quantified as an entropy difference:

$$\Delta H = H_{PMA}(T) - H_{noPMA}(T)$$

Where:

· ΔH is the entropy difference (stored in the 'Entropy_Diff' column of the CSV files)

· $H_{PMA}(T)$ is the entropy of target T in the PMA condition

· $H_{noPMA}(T)$ is the entropy of target T in the noPMA condition

A negative value of ΔH indicates that a target is more consolidated (lower entropy) in the PMA condition compared to noPMA, while a positive value indicates the target is more consolidated in the noPMA condition. The implementation used the entropy function from SciPy's scipy.stats module, which computes Shannon entropy according to this formula. In the code, this was applied to the normalized distribution of compounds across clusters for each target, with a minimum threshold of 5 compounds per target to ensure statistical reliability.
